# Hand, foot and mouth disease in China: evaluating an automated system for the detection of outbreaks

**DOI:** 10.2471/BLT.13.130666

**Published:** 2014-06-23

**Authors:** Zhongjie Li, Shengjie Lai, Honglong Zhang, Liping Wang, Dinglun Zhou, Jizeng Liu, Yajia Lan, Jiaqi Ma, Hongjie Yu, David L Buckeridge, Chakrarat Pittayawonganan, Archie CA Clements, Wenbiao Hu, Weizhong Yang

**Affiliations:** a Key Laboratory of Surveillance and Early-warning on Infectious Disease, Chinese Centre for Disease Control and Prevention, 155 Changbai Road, Changping District, Beijing, 102206, China.; b West China School of Public Health, Sichuan University, Chengdu, China.; c Sinosoft Company, Beijing, China.; d Department of Epidemiology, Biostatistics and Occupational Health, McGill University, Montreal, Canada.; e International Field Epidemiology Training Programme, Ministry of Public Health, Nonthaburi, Thailand.; f Research School of Population Health, The Australian National University, Canberra, Australia.; g School of Public Health and Social Work, Queensland University of Technology, Brisbane, Australia.

## Abstract

**Objective:**

To evaluate the performance of China’s infectious disease automated alert and response system in the detection of outbreaks of hand, foot and mouth (HFM) disease.

**Methods:**

We estimated size, duration and delay in reporting HFM disease outbreaks from cases notified between 1 May 2008 and 30 April 2010 and between 1 May 2010 and 30 April 2012, before and after automatic alert and response included HFM disease. Sensitivity, specificity and timeliness of detection of aberrations in the incidence of HFM disease outbreaks were estimated by comparing automated detections to observations of public health staff.

**Findings:**

The alert and response system recorded 106 005 aberrations in the incidence of HFM disease between 1 May 2010 and 30 April 2012 – a mean of 5.6 aberrations per 100 days in each county that reported HFM disease. The response system had a sensitivity of 92.7% and a specificity of 95.0%. The mean delay between the reporting of the first case of an outbreak and detection of that outbreak by the response system was 2.1 days. Between the first and second study periods, the mean size of an HFM disease outbreak decreased from 19.4 to 15.8 cases and the mean interval between the onset and initial reporting of such an outbreak to the public health emergency reporting system decreased from 10.0 to 9.1 days.

**Conclusion:**

The automated alert and response system shows good sensitivity in the detection of HFM disease outbreaks and appears to be relatively rapid. Continued use of this system should allow more effective prevention and limitation of such outbreaks in China.

## Résumé

**Objectif:**

Évaluer les résultats du système automatisé d'alerte et d'action pour les maladies infectieuses de la Chine en matière de détection des épidémies de maladie des mains, pieds et bouche (MMPB).

**Méthodes:**

Nous avons estimé la taille, la durée et le retard du signalement des épidémies de MMPB à partir des cas notifiés entre le 1er mai 2008 et le 30 avril 2010 et entre le 1er mai 2010 et le 30 avril 2012, c'est-à-dire avant et après l'intégration de la MMPB dans le système automatisé d'alerte et d'action. La sensibilité, la spécificité et la rapidité de la détection des aberrations dans l'incidence des épidémies de MMPB ont été estimées en comparant les détections automatisées aux observations du personnel des services de santé publique.

**Résultats:**

Le système d'alerte et d'action a enregistré 106 005 aberrations dans l'incidence de la MMPB entre le 1er mai 2010 et le 30 avril 2012 – une moyenne de 5,6 erreurs pour 100 jours dans chaque comté qui avait signalé la MMPB. Le système d'action avait une sensibilité de 92,7% et une spécificité de 95,0%. Le retard moyen entre le signalement du premier cas d'une épidémie et la détection de cette épidémie par le système d'action était de 2,1 jours. Entre les périodes de la première et de la seconde étude, la taille moyenne d'une épidémie de MMPB a diminué de 19,4 à 15,8 cas, et l'intervalle moyen entre le début et le signalement initial d'une telle épidémie au système de notification d'urgence des services de santé publique a diminué, passant de 10,0 à 9,1 jours.

**Conclusion:**

Le système automatisé d'alerte et d'action présente une bonne sensibilité en matière de détection des épidémies de MMPB et semble être relativement rapide. L'utilisation continue de ce système devrait permettre de prévenir et de limiter plus efficacement ces épidémies en Chine.

## Resumen

**Objetivo:**

Evaluar el rendimiento del sistema de alerta y respuesta automática a enfermedades infecciosas de China en la detección de brotes de la enfermedad boca-mano-pie (EBMP).

**Métodos:**

Calculamos la magnitud, la duración y la demora en la notificación de brotes de la enfermedad BMP a partir de casos notificados entre el 1 de mayo de 2008 y el 30 de abril de 2010, y entre el 1 de mayo de 2010 y el 30 de abril 2012, antes y después de que el sistema de alerta y respuesta automáticas incluyera la enfermedad BMP. Se estimó la sensibilidad, especificidad y oportunidad de la detección de aberraciones en la incidencia de los brotes de enfermedad BMP mediante la comparación de las detecciones automáticas con las observaciones del personal de salud pública.

**Resultados:**

El sistema de alerta y respuesta registró 106 005 aberraciones en la incidencia de la EBMP entre el 1 de mayo de 2010 y el 30 de abril de 2012 - una media de 5,6 aberraciones por cada 100 días en cada condado que notificó dicha enfermedad. El sistema de respuesta tuvo una sensibilidad del 92,7 % y una especificidad del 95,0 %. La demora media entre la notificación del primer caso de un brote y la detección de ese brote por el sistema de respuesta fue de 2,1 días. Entre el primer y el segundo período de estudio, las dimensiones medias de un brote de EBMP disminuyeron de 19,4 a 15,8 casos, y el intervalo medio entre el comienzo de un brote y el primer informe en el sistema de notificación de emergencia de salud pública se redujo de 10,0 a 9,1 días.

**Conclusión:**

El sistema de alerta y respuesta automática muestra una buena sensibilidad en la detección de los brotes de EBMP y parece ser relativamente rápido. El uso continuado de este sistema debería permitir una prevención y limitación más eficaces de dichos brotes en China.

## ملخص

**الغرض:**

تقييم أداء نظام الإنذار والاستجابة الآلي للأمراض المعدية في اكتشاف فاشيات مرض اليد والقدم والفم في الصين.

**الطريقة:**

قمنا بتقدير الحجم والمدة والتأخير في الإبلاغ عن فاشيات مرض اليد والقدم والفم من الحالات التي تم الإخطار بها بين 1 أيار/مايو 2008 و30 نيسان/أبريل 2010 وبين 1 أيار/مايو 2010 و30 نيسان/أبريل 2012، قبل إدراج داء اليد والقدم والفم في الإنذار والاستجابة الآلية وبعده. وتم تقدير حساسية اكتشاف الانحرافات ونوعيتها وتوقيتها في معدل الإصابة بفاشيات مرض اليد والقدم والفم عن طريق مقارنة الاكتشافات والملاحظات الآلية من قبل موظفي الصحة العمومية.

**النتائج:**

سجل نظام الإنذار والاستجابة 106005 انحرافاً في معدل الإصابة بمرض اليد والقدم والفم بين 1 أيار/مايو 2010 و30 نيسان/أبريل2012 - المتوسط 5.6 انحرافاً لكل 100 يوم في كل مقاطعة أبلغت عن مرض اليد والقدم والفم. وكانت حساسية نظام الاستجابة 92.7 % ونوعيته 95.0 %. وكان متوسط التأخير بين الإبلاغ عن الحالة الأولى لإحدى الفاشيات واكتشاف تلك الفاشية عن طريق نظام الاستجابة 2.1 يوم. وانخفض متوسط حجم فاشية مرض اليد والقدم والفم بين الفترتين الدراسيتين الأولى والثانية من 19.4 حالة إلى 15.8 حالة وانخفض متوسط الفاصل بين بداية هذه الفاشية والإبلاغ الأولي عنها إلى نظام الإبلاغ عن حالات الطوارئ في الصحة العمومية من 10.0 إلى 9.1 يوماً.

**الاستنتاج:**

يشير نظام الإنذار والاستجابة الآلي إلى حساسية جيدة في اكتشاف فاشيات مرض اليد والقدم والفم ويبدو أنه سريع نسبياً. وينبغي أن يسمح الاستخدام المتواصل لهذا النظام بزيادة فعالية الوقاية وتقييد هذه الفاشيات في الصين.

## 摘要

**目的:**

评价中国传染病自动预警与响应系统在探测手足口疾病（HFM）爆发方面的表现。

**方法:**

我们利用2008年5月1日和2010年4月30日之间以及2010年5月1日和2012年4月30日之间（即自动预警与响应系统将手足口病纳入之前和之后）报告的病例估算了手足口病病爆发的规模、持续时间和报告延迟时间。将预警与响应系统自动探测的结果与公共卫生工作人员的观察结果进行比较，评估该系统用于探测手口足病爆发的灵敏度、特异度和及时性。

**结果:**

在2010年5月1日至2012年4月30日期间，预警和响应系统发出了106005条预警信号，——在每个有手足口病病例报告的县，平均每100天产生5.6条手足口病预警信号。响应系统敏感度为92.7%，特异度为95.0%。从爆发的第一例病例报告至自动预警与响应系统探测到爆发之间的平均时间间隔是2.1天。在自动预警与响应系统将手足口病纳入之前和之后，手足口病爆发的平均规模从19.4例下降到15.8例，从爆发首例病例发病至向突发公共卫生事件报告系统进行报告之间的平均时间间隔从10.0天下降到9.1天。

**结论:**

自动预警与响应系统在探测手足口病爆发方面具有良好的灵敏度和及时性。持续使用该系统应该能够更有效地预防和控制中国手足口病的爆发。

## Резюме

**Цель:**

Оценить эффективность автоматизированной системы оповещения и реагирования в Китае, используемой для обнаружения вспышек вирусной пузырчатки полости рта и конечностей (HFM — от англ. hand, foot and mouth disease).

**Методы:**

Размер, продолжительность вспышек и задержка сообщений о вспышках HFM оценивались на основе случаев, зарегистрированных в период с 1 мая 2008 года по 30 апреля 2010 года и в период с 1 мая 2010 года по 30 апреля 2012 года, как до, так и после начала использования системы автоматического оповещения и реагирования о данном заболевании. Чувствительность, специфичность и своевременность обнаружения отклонений в уровне заболеваемости HFM оценивались путем сравнения данных автоматизированной системы обнаружения с наблюдения специалистов общественного здравоохранения.

**Результаты:**

Системой оповещения и реагирования было зарегистрировано 106 005 отклонений в уровне заболеваемости HFM в период с 1 мая 2010 года по 30 апреля 2012 года, что составило в среднем 5,6 отклонений на 100 дней в каждом округе, сообщившем о случаях заболевания HFM. Чувствительность и специфичность системы реагирования составляли 92,7% и 95,0% соответственно. Средняя задержка между сообщением о первом случае вспышки заболевания и выявлением данной вспышки системой реагирования составила 2,1 дня. Между первым и вторым периодами исследования средний размер вспышки HFM уменьшился с 19,4 до 15,8 случаев, а средний интервал между началом вспышки и первоначальным сообщением о данной вспышке в систему аварийной отчетности здравоохранения уменьшился с 10,0 до 9,1 дней.

**Вывод:**

Автоматизированная система оповещения и реагирования продемонстрировала хорошую чувствительность при обнаружении вспышек вирусной пузырчатки полости рта и конечностей и относительно высокую скорость реагирования. Дальнейшее использование этой системы должно обеспечить более эффективное предотвращение и ограничение вспышек данного заболевания в Китае.

## Introduction

To improve control of infectious disease outbreaks, it is critical to establish early detection and warning systems. In recent decades, technological advances in computing and communication and mathematical aberrancy-detection algorithms have been applied to high-volume data sets, to generate alerts and draw the attention of epidemiologists to statistical anomalies that may indicate a localized outbreak or the elevated risk of such an outbreak.^1^^–^^3^ Several national public health agencies have successfully developed and operated automated early warning systems for the prompt detection of disease outbreaks.^4^^–^^8^ Some epidemiologists have simulated outbreaks to evaluate the performance of such systems and the associated outbreak-detection algorithms.^9^^,^^10^ However, there have been few prospective evaluations of the performance of early warning systems in operational settings.^11^^,^^12^

In April 2008, a web-based automated system for the early detection of – and rapid response to – outbreaks of infectious disease was implemented across China.^13^ This system – the China infectious disease automated alert and response system (hereafter referred to as the response system) – was developed by the Chinese Centre for Disease Control and Prevention, with the support of the Chinese Ministry of Health and the World Health Organization. The response system was based on surveillance data on dozens of notifiable diseases and on several statistical algorithms for the automated and routine detection of aberrations in such data, at county level, that might indicate the early stages of potential outbreaks.

Although hand, foot and mouth (HFM) disease can be caused by serotypes of several enteroviruses, it is most frequently caused by coxsackie virus A16 and human enterovirus 71. Most affected people develop only mild symptoms but some cases may result in serious and even fatal complications.^14^^–^^16^ In China, HFM disease is frequently detected in children aged less than five years^17^ and there have been over a million cases of the disease, including hundreds of fatal cases, reported annually over recent years.^18^^,^^19^

In this study, we aimed to evaluate the performance of the response system by analysing the sensitivity, specificity and timeliness in the detection of HFM disease outbreaks. We also wished to evaluate the response system’s effectiveness by comparing the size and duration of HFM disease outbreaks – and the post-onset delay in reporting such outbreaks – before and after HFM disease was included in the response system.

## Methods

### Case reporting system

All HFM disease cases that occurred in China after May 2008 – when HFM disease became a notifiable disease in China^20^ – should have been reported, by attending clinicians, via the nationwide notifiable infectious diseases reporting information system (hereafter referred to as the case reporting system). This system enables health-care institutes across China to report information on each case of a notifiable infectious disease rapidly, via the Internet, to the Chinese Centre for Disease Control and Prevention. For our study, we used the information on each laboratory-confirmed or clinically diagnosed case of HFM disease that was reported to the case reporting system between 1 May 2008 and 30 April 2012.

### Automated detection of outbreaks

**Fig. 1:**
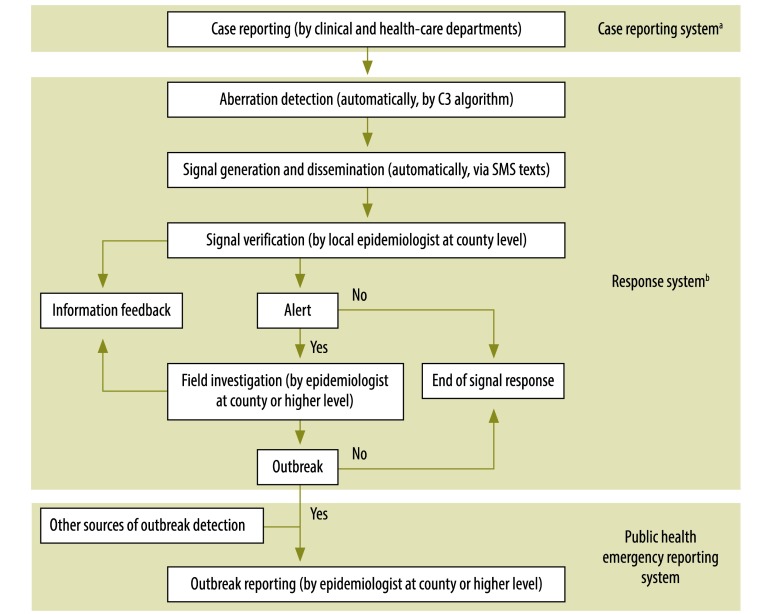
The operational flow of information on hand, foot and mouth disease to and from the response system^a^

^a^ China Infectious Disease Automated Alert and Response System.

^b^ Notifiable Infectious Diseases Reporting Information System.

Currently, the automated alert and response system searches the data collected in the case reporting system for aberrations in the incidence of HFM disease and another 29 notifiable infectious diseases.^13^ HFM disease has only been included in the response system since 1 May 2010. In the response system, an aberration in incidence at county level leads to the automated generation of a warning signal and that signal’s dissemination to the relevant county-level Centre for Disease Control and Prevention. Each signal is then investigated further by epidemiologists in the specific county (Fig. 1).

#### Aberration detection

Aberration detection of HFM disease outbreak in the response system is based on the C3 algorithm of the early aberration reporting system developed by the United States Centers for Disease Control and Prevention.^2^^,^^9^^,^^21^^–^^24^ C3 compares the count of cases in the current day – day 0 – with the corresponding mean count and standard deviation for seven earlier days – days −9 to −3. If the calculated value of C3 surpasses a preset threshold, a warning signal is generated. Following the advice of senior epidemiologists and statisticians in the response system’s research group, the preset threshold was given a value of 1.3 for HFM disease in May 2010. This value took estimates of the response system’s general sensitivity, timeliness, specificity and positive predictive value in outbreak detection into account.

#### Signal generation and dissemination

Once a day, the response system searches for aberrations in the county-level incidence of HFM disease. Any warning signals generated are then automatically disseminated via short message service texts sent to the mobile phones of designated staff in the Centres for Disease Control and Prevention in the relevant counties.^13^^,^^25^

#### Investigation and feedback

A health-care professional who receives a warning signal as a text message is expected to review the HFM disease cases that triggered the signal, further assess the possibility of an outbreak – by integrating information from other sources, such as information collected by direct contact with the reporting clinical and health-care agencies and – if there then seems to be a real threat of an outbreak (which meant that the warning signal became an alert signal) – conduct a field investigation.^13^^,^^25^ If an HFM disease outbreak is confirmed after field investigation, it should be reported to the public health emergency reporting system.

The health-care professionals who receive warning signals are expected to complete two simple, web-based forms, as soon as possible, so that details of how the professionals proceeded with signal verification and – if appropriate – field investigation can be viewed promptly by epidemiologists at higher levels.^13^ In this way, high-level epidemiologists can carefully monitor and assess the risk of outbreak spread.

#### Reporting confirmed outbreaks

The Chinese public health emergency reporting system was initiated in 2004, to record outbreaks of infectious diseases identified by local epidemiologists. Aside from the procedures that form part of the response system, staff from local health departments are instructed to conduct a field investigation if, within 1 week, at least five HFM disease cases occur in the same setting – e.g. kindergarten or school – or at least three cases of the disease occur in the same village or community. Any outbreak confirmed by a field investigation should be reported to the public health emergency reporting system.^20^^,^^26^

### Evaluating the response system’s effectiveness

The main objectives of our study were to evaluate the response system’s capacity for identifying HFM disease outbreaks and the response system’s impacts on the mean size and duration of an HFM disease outbreak and on the mean delays in the recording of an HFM disease outbreak to the public health emergency reporting system. The HFM disease outbreaks recorded in the public health emergency reporting system were used as the gold standard in our estimations of the response system’s sensitivity, specificity and timeliness. The number of cases detected was used as the measure of the size of an outbreak. The number of days between the onset of symptoms in the first and last known cases that were related to the outbreak was used as the estimate of outbreak duration. Sensitivity was estimated by dividing the number of HFM disease outbreaks detected by the response system, by the corresponding number of such outbreaks recorded in the public health emergency reporting system.^9^^,^^27^ Specificity was estimated by dividing the number of non-outbreak days on which no warning signal was generated for HFM disease – by the response system – by the total number of non-outbreak days. Time to detection was defined as the interval between the first case related to the outbreak being reported to the reporting system and the generation of the first warning signal about the outbreak by the response system.^27^ Time from detection to report was defined as the interval between the generation of the first warning signal about the outbreak by the response system and the report of the outbreak to the public health emergency reporting system. Time to report – which was investigated both before and after the response system was implemented – was defined as the interval between symptom onset in the first case related to the outbreak and the report of the outbreak to the public health emergency reporting system.

The mean size, duration and time to report of an HFM disease outbreak were estimated for the period 1 May 2008–30 April 2010 – i.e. before HFM disease was covered by the response system – and for the period 1 May 2010–30 April 2012 – i.e. after HFM disease was included in the response system’s remit.

### Statistical analyses

We used Pearson’s *χ^2^* test to evaluate the significance of the response system’s sensitivity in the detection of HFM disease outbreaks in three size categories: 3–10, 11–20 and more than 20 cases. Time to detection was investigated by one-way analysis of variance. Student’s *t*-test was used to examine whether the mean size, duration and time to report of outbreaks were significantly different before and after HFM disease was included in the response system. All analyses were implemented in version 2.14.1 of the R statistical software package (R Foundation for Statistical Computing, Vienna, Austria).

## Results

Between 1 May 2008 and 30 April 2012, 5 471 108 cases and 1209 outbreaks of HFM disease were reported in China (Table 1). The number of HFM disease cases per month ranged from 7512 cases in January 2009 to 353 104 cases in May 2010, with a mean value of 113 981 (95% confidence interval, CI: 87 444–140 186). Over this period, HFM disease incidence showed marked seasonality, with a major peak – comprising almost half of all cases –in April–June and a smaller secondary peak – comprising 18.0% of cases – in September–November. Reported outbreaks, warning signals and alerts showed a similar seasonal pattern.

**Table 1:** Outbreaks of hand, foot and mouth disease in China, 2008–2012

Indicator	Period				
	1 May 2008–30 April 2009	1 May 2009–30 April 2010	1 May 2010–30 April 2011	1 May 2011–30 April 2012	Overall
**Cases**					
Cases reported in the case reporting system^a^	757 141	1 256 320	1 576 918	1 880 729	5 471 108
Outbreaks recorded by the public health emergency reporting system	211	380	298	320	1 209
Ratio of all reported cases^b^ to those recorded in the public health emergency reporting system	3 588:1	3 306:1	5 292:1	5 877:1	4 525:1
No. of cases related to outbreaks	4 077	7 376	4 795	4 956	21 204
Ratio of all reported cases^b^ to cases related to outbreaks	1:186	1:170	1:329	1:379	1:258
**Signals**					
Warning signals generated by the response system^c^	–	–	48 916	57 089	106 005
Ratio of all cases to warning signal^b^	–	–	32:1	33:1	33:1
Alerts recorded in response system^c^	–	–	1 117	1 244	2 361
Ratio of warning signals to alerts^b^	–	–	44:1	46:1	45:1
Detected outbreaks	–	–	278	295	573
Ratio of alerts to detected outbreaks^b^	–	–	4:1	4:1	4:1

^a^ Notifiable Infectious Diseases Reporting Information System.

^b^ Rounded to an integer.

^c^ China Infectious Disease Automated Alert and Response System.

The number of outbreaks reported per year ranged from 211 for the period 1 May 2008–30 April 2009 to 380 for the period 1 May 2009–30 April 2010. Between 1 May 2010 and 30 April 2012, 106 005 warning signals in a total of 2608 counties were generated by the response system for HFM disease (Table 1). This represents a mean of 5.6 such signals every 100 days in each of the counties that had at least one signal. Initial verification indicated that 2361 (2.2%) of the signals merited being raised to alert status and field investigation. Field investigation of the response system’s signals led to 573 HFM disease outbreaks being confirmed. The response system received the initial verification results for 94 920 (89.5%) of the signals within 24 h.

As 618 HFM disease outbreaks were recorded in the public health emergency reporting system in the period when 573 such outbreaks were identified in the response system, the overall sensitivity of the response system in the detection of HFM disease outbreaks was 92.7% (Table 2). The response system’s sensitivity was significantly higher for large outbreaks involving more than 20 cases than for small outbreaks that involved no more than 10 cases (99.3% versus 84.6%; *P* < 0.001). In the detection of HFM disease outbreaks, the overall specificity of the response system was 95.0% (19 74 324/2 078 361) and the overall mean time to detection was 2.1 days (95% CI: 1.8–2.3). The mean time to detection was 1.7 days for outbreaks that involved no more than 10 cases but 2.7 days for outbreaks that involved more than 20 cases. The mean time from detection to report in the public health emergency reporting system was 4.5 days (95% CI: 4.1–5.0).

**Table 2:** Detection of outbreaks of hand, foot and mouth disease in China, 1 May 2010–30 April 2012

No. of cases in outbreak	No. of outbreaks			Performance of response system^a^	
	Reported in public health emergency reporting system	Detected by response system^a^		Sensitivity, %^b^	Mean time to outbreak detection, days^c^ (95% CI)
3–10	156	132		84.6	1.7 (1.3–2.1)
11–20	326	306		93.9	1.9 (1.7–2.2)
> 20	136	135		99.3	2.7 (1.9–3.5)
Overall	618	573		92.7	2.1 (1.8–2.3)

CI: confidence interval.

^a^ China Infectious Disease Automated Alert and Response System.

^b^ Values differ significantly according to size of outbreak (*P* < 0.001).

^c^ The time between the reporting of the first known case of an outbreak and the response system’s generation of the first warning signal about that outbreak. Values do not differ significantly according to size of outbreak (one-way analysis of variance; *P* = 0.28).

In our investigation of the data recorded before HFM disease was included in the response system, the mean size (*P* = 0.982), duration (*P* = 0.572) and time to report (*P* = 0.358) of the HFM disease outbreaks detected between 1 May 2008 and 30 April 2009 were similar to those of the outbreaks detected in the following 12 months. Similarly, in our investigation of the data recorded after HFM disease was included in the response system, the mean size (*P* = 0.443), duration (*P* = 0.370) and time to report (*P* = 0.840) of the HFM disease outbreaks detected between 1 May 2010 and 30 April 2011 were similar to those of the outbreaks detected in the following 12 months. The outbreaks recorded in the two years immediately after HFM disease was included in the response system were generally smaller than those recorded over the previous two years, with mean sizes of 15.8 and 19.4 cases, respectively (Table 3). The mean size of outbreaks that involved more than 20 cases was significantly less in the two years immediately after HFM disease was included in the response system than the corresponding value for the previous two years (29.2 versus 55 cases; *P* = 0.015).

**Table 3:** Size, duration and reporting times of hand, foot and mouth (HFM) disease outbreaks before and after response system^a^ application, China, 2008–2012

No. of cases before/after inclusion of HFM disease in response system^a^	Outbreaks of HFM disease reported to public health emergency reporting system			
	No. reported	Mean size, cases (95% CI)	Mean duration, days (95% CI)	Mean time to report, days (95% CI)
**Before inclusion^b^**				
3–10	161	6.7 (6.3–7.1)	9.1 (8.2–10.0)	8.1 (7.4–8.7)
11–20	328	14.5 (14.2–14.8)	14.0 (13.1–14.9)	10.1 (9.5–10.7)
> 20	102	55.0 (34.3–75.8)	28.7 (24.4–32.9)	12.7 (11.1–14.3)
Overall	591	19.4 (15.6–23.2)	15.2 (14.1–16.2)	10.0 (9.5–10.5)
**After inclusion^c^**				
3–10	156	6.4 (5.9–6.8)	8.4 (7.6–9.2)	7.3 (6.8–7.8)
11–20	326	14.7 (14.4–15.0)	14.0 (13.2–14.7)	9.4 (8.9–9.8)
> 20	136	29.2 (27.2–31.1)^d^	26.0 (23.5–28.5)	10.5 (9.5–11.5)^e^
Overall	618	15.8 (15.0–16.5)	15.2 (14.4–16.1)	9.1 (8.7–9.5)^f^

CI: confidence interval.

^a^ China Infectious Disease Automated Alert and Response System;

^b^ For the period 1 May 2008–30 April 2010.

^c^ For the period 1 May 2010–30 April 2012.

^d^ Significantly lower than corresponding value for the study period before HFM disease was included in CIDARS (*P* = 0.015).

^e^ Significantly lower than corresponding value for the study period before HFM disease was included in CIDARS (*P* = 0.020)

^f^ Significantly lower than corresponding value for the study period before HFMD disease was included in CIDARS (*P* = 0.004).

The overall mean duration of an HFM disease outbreak was estimated to be 15.2 days for the study periods before and after HFM disease was included in the response system. However, the mean duration of outbreaks that involved more than 20 cases fell from 28.7 days in the two years before HFM disease was included in the response system to 26.0 days in the following two-year period. The corresponding falls in the mean number of days taken to report an HFM disease outbreak of any size – from 10.0 to 9.1 (*P* = 0.004) – and an HFM disease outbreak that involved more than 20 cases – from 12.7 to 10.5 (*P* = 0.020) – were significant.

## Discussion

Our observations indicate that the response system had good sensitivity and specificity in the detection of HFM disease outbreaks and could lead to a reduction in the eventual size of an outbreak – by shortening the reporting time and so permitting an earlier response.

Our results are consistent with previous research that has found the C3 algorithm to be useful for the detection of aberrancy in the incidence of influenza, bacillary dysentery, HFM disease and other diseases.^22^^,^^23^^,^^27^ We found that the response system’s sensitivity in detecting outbreaks of HFM disease that became relatively large – i.e. 99.3% for outbreaks with more than 20 cases – was significantly higher than that for outbreaks that remained small – i.e. 84.6% for outbreaks with no more than 10 cases. Perhaps the outbreaks that grow large expand relatively rapidly and quickly present a large enough deviation from the baseline value for incidence to be easily detected. However, we made no attempt to investigate how responses to the detected outbreaks affected their final size. Overall, 45 HFM disease outbreaks – that were confirmed by health professionals at a time when HFM disease was included in the response system – were not detected by the response system. All 45 remained relatively small and occurred in kindergartens, elementary schools or rural villages. Efforts should be made to increase the sensitivity of the response system – e.g. by using high-resolution spatial detection methods^28^^–^^30^ – to improve the prompt detection of outbreaks while they are small.

Although use of a C3 threshold of 1.3 resulted in good sensitivity, specificity and timeliness in the response system’s detection of HFM disease outbreaks, it also resulted in a low positive predictive value. The health professionals who checked the data decided that only 2.2% of the warning signals that the response system generated for HFM disease merited field investigation. One cause of the low positive predictive value is that almost all of the HFM disease cases seen in China – over 99.6% according to the data that we analysed – are sporadic and never form part of an outbreak. A temporal cluster of sporadic cases may easily trigger a false-positive warning signal in the response system. Such false signals need to be reduced by optimizing the algorithms and thresholds used for outbreak detection – perhaps according to the relevant baseline incidence of HFM disease.^31^ The procedures for the verification of warning signals at county level also need to be simplified, to reduce the detrimental effects of so many false-positive signals on the morale and workloads of health professionals.

The early detection of potential outbreaks is important in minimizing the impact of HFM disease.^19^ Inclusion of HFM disease in the national response system cut the time taken to report an outbreak of the disease by almost a day. Since the corresponding warning signals were generated a mean of 4.5 days before the outbreaks were reported, there is clearly scope to further reduce the mean time taken to report a confirmed outbreak. Early detection allows the early implementation of outbreak control measures – such as health surveys for the detection of other cases, case isolation, disinfection of affected settings, health education, promotion of hand hygiene, and closure of affected classes or schools – as well as the early treatment of cases and the prevention of the more severe complications of HFM disease.

One limitation of our study was that, for calculating the sensitivity, specificity and timeliness of the response system, we used the outbreaks reported to the public health emergency reporting system as the gold standard. It seems likely that some outbreaks of HFM disease are either never recorded by the public health emergency reporting system or are reported a long time after they have occurred. These issues need investigation. However, at the time of our study, we believed that the public health emergency reporting system was the best-functioning system for the collection of data on HFM disease outbreaks in China.

Our findings demonstrate that – if well designed and operated – an automated early warning system for outbreaks of infectious disease can help local epidemiologists identify outbreaks rapidly, thereby facilitating the prevention of outbreak spread. The response system’s design framework and methods could provide a useful example for institutes of public health in many countries.
